# Persistent Urogenital Sinus Leading to Hydrometrocolpos in a Female Child With Features of McKusick-Kaufman Syndrome

**DOI:** 10.7759/cureus.61957

**Published:** 2024-06-08

**Authors:** Janhavi V Thorat, Sampada Tambolkar

**Affiliations:** 1 Pediatrics, Dr. D. Y. Patil Medical College, Hospital & Research Centre, Dr. D. Y. Patil Vidyapeeth (Deemed to be University) Pimpri, Pune, IND

**Keywords:** foot polydactyly, overlapping syndromes, hydroureteronephrosis, genetic syndromes, urogenital sinus

## Abstract

Persistent urogenital sinus (PUGS) presents as a solitary abnormality or is in association with syndromes, such as congenital adrenal hyperplasia (CAH), VACTERL association (common abbreviation for vertebral defects, anal atresia, cardiac defects, tracheoesophageal fistula, renal anomalies, and limb abnormalities), Bardet-Beidl syndrome, McKusick-Kaufman syndrome (MKS), and Townes-Brocks syndrome, to name a few. Those affected usually have overlapping phenotypic features of two or more syndromes. Because such children may grow up to be intellectually challenged with multiple other anomalies including gonadal hyperplasia, congenital heart defects, and sensorineural hearing loss, antenatal diagnosis becomes important. Moreover, those who survive into childhood may need a holistic approach to improve their quality of life. This is a rare case of an eight-year-old female child who is a postnatally diagnosed case of congenital heart disease, urogenital sinus with polydactyly, and bilateral hydroureteronephrosis at birth and who is now showing features of multiple overlapping syndromes.

## Introduction

Persistent urogenital sinus (PUGS) is a rare congenital anomaly with an incidence of six per 100,000 women [1,2]. It is a tract joining the vagina and the urinary system. It may present as a solitary finding or in association with congenital adrenal hyperplasia (CAH), where its incidence is 1:500 [3], or as a part of syndromes, such as VACTERL (common abbreviation for vertebral defects, anal atresia, cardiac defects, tracheoesophageal fistula, renal anomalies, and limb abnormalities) [4]. Postaxial polydactyly associated with genital abnormalities is found only in a handful of syndromes, such as Townes-Brocks syndrome [5], McKusick-Kaufman syndrome (MKS) [6], and Bardet-Biedl syndrome [7] to name a few. Vaginoplasty with urogenital sinus mobilization is now the preferred mode of treatment in cases of PUGS. It is recommended to perform corrective procedures before two years of age [8]. Many of these syndromes overlap phenotypically. One such example is the phenotypical overlap of Bardet-Biedl syndrome and MKS [9]. This makes a single diagnosis difficult. Hence, a high suspicion of syndromic associations should be maintained to screen for and treat associated anomalies.

## Case presentation

A 27-year-old woman gave birth to dichorionic diamniotic female twins at 32 weeks of gestation via caesarian section performed due to the premature rupture of membranes in the year 2016. The last available scan was done at an estimated fetal gestational age of 20 weeks, which showed no abnormality in either fetus. In the last scan, the estimated fetal weight of twin A (affected twin) was approximately 268 grams while that of twin B (unaffected twin) was about 313 grams. They were reported to have mild but insignificant discordance in intrauterine growth. At birth, twin B had a birth weight of 1400 grams (22nd centile) with APGAR scores of six at one minute and seven at 5 minutes. She was endotracheally intubated and ventilated. At four days of age, ultrasonography of the abdomen and pelvis was performed due to abdominal distension, which revealed a grossly distended cystic mass seemingly arising from and occupying the pelvis and extending up to the epigastrium with a volume of 100 ml and a wall thickness of 2 mm. A lot of sludge was commented to be seen. The report stated a possibility of bladder outlet obstruction leading to obstructive uropathy. Considering a normal anomaly scan, this feature must have grown rapidly after the 20th-week mark. Keeping in mind a possibility of bladder outlet obstruction causing obstructive uropathy, cystoscopy and CT scan were planned after stabilization.

At five days of age, her urine examination showed 60-70 pus cells with three to four red blood cells with a urine culture positive for *Enterobacter cloacae* complex, and she was treated with intravenous amikacin for the same. Urine cultures on the 24th day of life showed no growth of organisms. On the 30th day of life, her blood and urine cultures were positive for *Escherichia coli* and *Klebsiella pneumoniae*, respectively, for which she was further treated with intravenous injection meropenem and injection amikacin for 14 days. An echocardiography of the heart performed to rule out other congenital malformations revealed a ventricular septal defect. Cystoscopy was done at two months of life and a diagnosis of congenital urogenital sinus with large hydrometrocalpos was made with a plan for operative correction. Apart from this, during her course in the neonatal intensive care unit, she also developed neonatal cholestasis (treated with ursodeoxycholic acid) and had a patent ductus arteriosus treated with oral paracetamol and zone 2 retinopathy of prematurity for which she had laser photocoagulation done. She also had one episode of abnormal movements during her stay in NICU for which no long-term medication was started.

**Figure 1:**
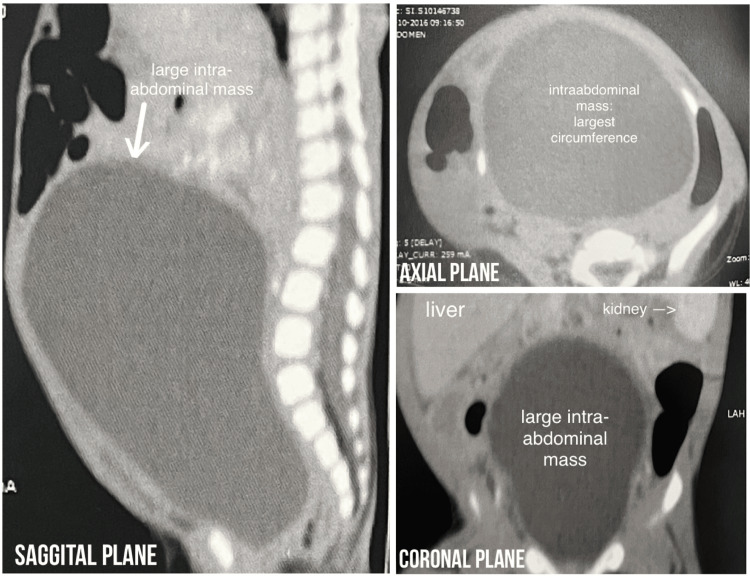
Computed tomography (CT) scan of the patient done in the fourth month of life. Computed tomography (CT) scan of the abdomen and pelvis revealed a large cystic lesion measuring 10 x 6 x 7 cm in the craniocaudal, transverse, and anteroposterior axis, displacing both the urinary bladder and the uterus anteriorly and cranially while also displacing mesenteric vessels and small bowel loops towards its periphery with minimal fluid in the endometrial cavity. This cystic lesion was also causing a mass effect on the distal pelvic ureters, hence causing bilateral hydronephrosis and hydroureters.

At four months of life, a computed tomography scan (CT scan) of the abdomen and pelvis (Figure 1) revealed a large cystic lesion measuring 10 x 6 x 7 cm in the craniocaudal, transverse, and anteroposterior axis, displacing both the urinary bladder and the uterus anteriorly and cranially while also displacing mesenteric vessels and small bowel loops toward its periphery with minimal fluid in the endometrial cavity. This cystic lesion was also causing a mass effect on the distal pelvic ureters, hence causing bilateral hydronephrosis and hydroureters.

**Figure 2:**
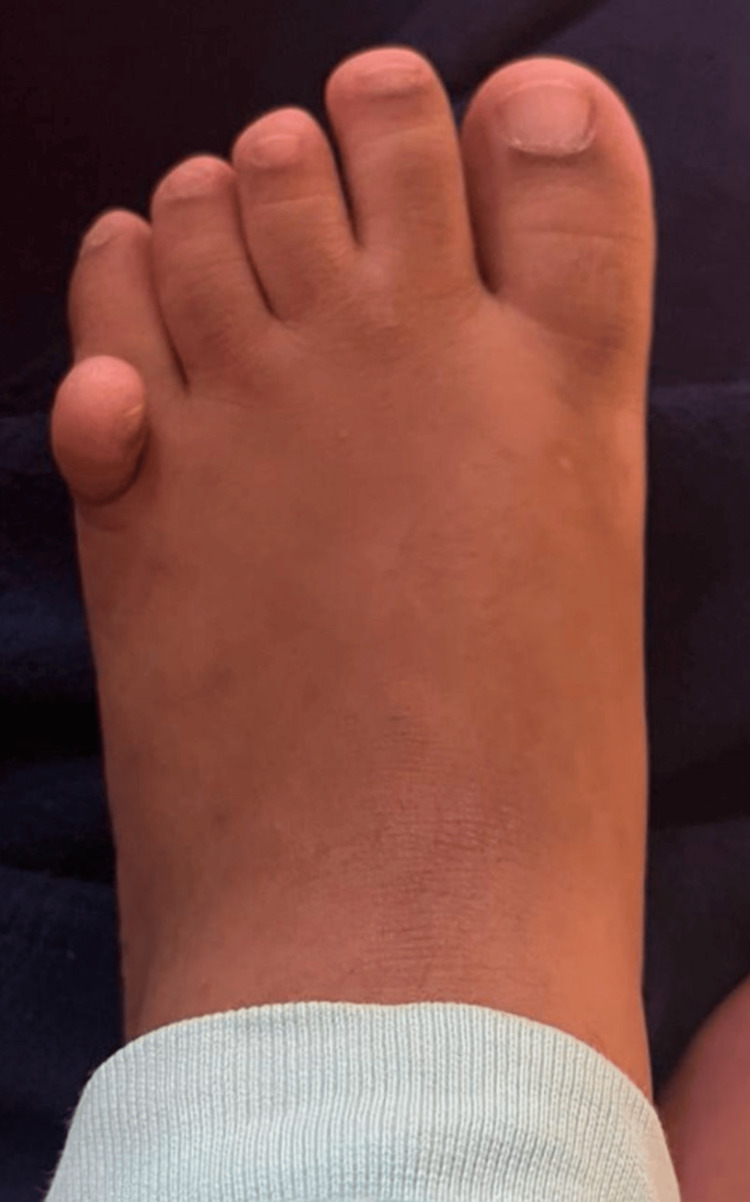
Polydactyly seen in the left limb.

An exploratory laparotomy with suprapubic cystostomy with adhesiolysis was performed for the same at five months of life. At 13 months of life, she further underwent a urethrovaginoplasty with total urogenital sinus mobilization. Throughout her history of hospitalisation, she never had hyponatremia, hyperkalemia, or hypoglycemia (features typical of CAH). At 15 months of life, an L,L-ethylenedicysteine scan (EC scan) of the kidneys showed a split function of the left kidney being 41.2% while the right kidney being 58.8%. This suggests a small but satisfactorily functioning left kidney. She was noted to have multiple features suggesting a syndromic etiology, including tapering fingers of hands, post-axial polydactyly along with partial syndactyly of the second and third toes in the left lower limb (Figure 2), small anteriorly malrotated ears (brainstem evoked response audiometry done at six months of age revealed normal hearing), up-slanting eyes, and microphthalmia.

**Figure 3:**
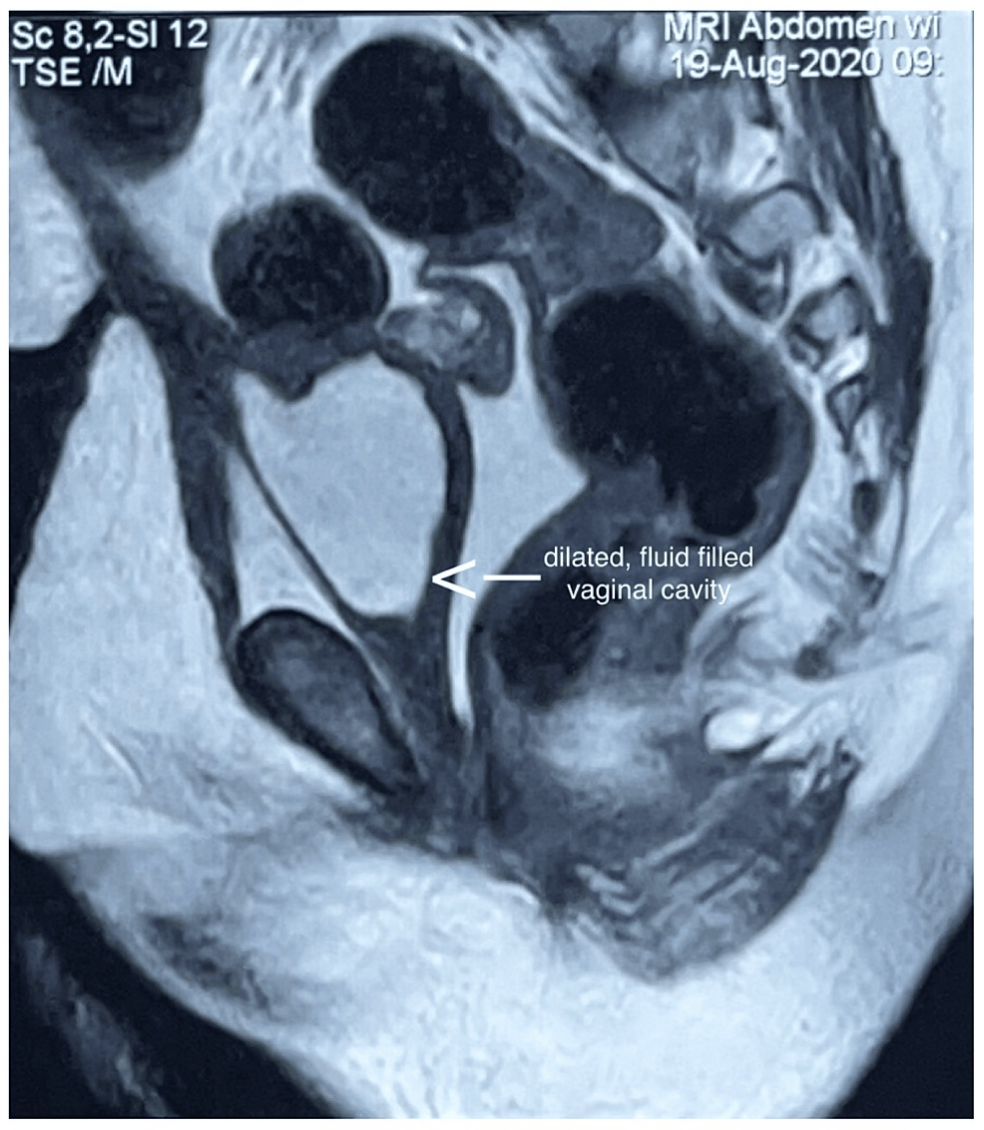
Magnetic resonance imaging scan of the abdomen and pelvis done at four years of age showing a dilated, fluid-filled vaginal cavity.

**Figure 4:**
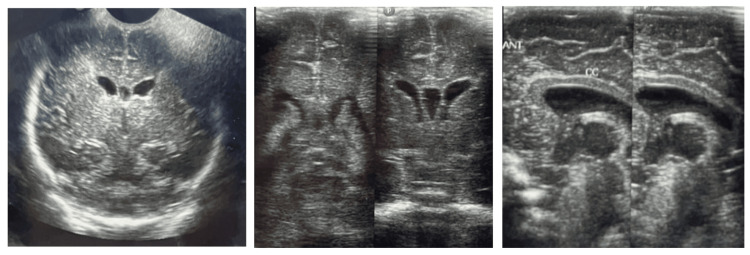
Ultrasonography of the brain done during the neonatal period showed no evidence of intraventricular hemorrhage.

Her latest magnetic resonance imaging (MRI) scan of the abdomen and pelvis done at the age of four years showed a dilated fluid-filled vaginal cavity (Figure 3). Her ultrasonography of the abdomen and pelvis done in the year 2023 shows the same findings with an additional finding of bilateral mild fullness of the pelvicalyceal system and ureters (an improvement from earlier findings). At five years of age, she started having right focal seizures with post-ictal drowsiness five to six times a day, even during sleep. Parents also noticed behavioral disturbances like aggression and early irritability. She also started developing central obesity at this age. Pendular nystagmus was also noted on examination. Electroencephalogram showed bilateral focal centro-temporal and parietal epileptiform activity. She was started on oral oxcarbazepine and risperidone for the same. Tablet clobazam was added to control iatrogenic tremors. MRI of the brain showed changes in periventricular leukomalacia, prominent bilateral lateral ventricles, and mild cerebral atrophy, even though brain ultrasounds done during the NICU stay showed no evidence of intraventricular hemorrhage (Figure 4). Developmental delay was present in all domains for which she underwent evaluation with the Developmental Assessment Scale for Indian Infants (DASII) and the Vineland Social Maturity Scale (VSMS). According to DASII, at a chronological age of 64 months, her development age was 13.2 months. According to the VSMS, her social quotient was 70, suggestive of low socio-adaptive functioning. Currently, she attends behavioral therapy with a special educator. She weighs 35 kg with a height of 116.8 cm, which makes her BMI 25.7 falling in the obese category (above the 27th percentile of adult equivalent). She has evident central obesity despite a healthy diet. 

## Discussion

PUGS is a rare congenital anomaly with an incidence of six per 100,000 women [1,2], which most commonly presents in association with CAH (incidence of 1:500) [3]. It may or may not be associated with other anomalies and syndromes like VACTERL [4], Townes-Brocks syndrome [5], MKS [6], and Bardet-Biedl syndrome [7]. Patients may present with overlapping phenotypes, hence making the diagnosis difficult [9]. Moreover, all the syndromes associated with PUGS are rare and may hence easily evade diagnosis. As the problems of monetary restrictions and limited accessibility to services such as genetic testing prevail in low-income countries like India [10], physicians may have to primarily rely on clinical assessment and minimal investigations to make a diagnosis.

This case has findings of intrauterine fetal growth restriction, postnatally diagnosed PUGS, hydrometrocolpos (HMC), ventricular septal defect, developmental delay in all domains, central obesity, short stature, seizures, pendular nystagmus, tapering fingers of hands, post-axial polydactyly in the left lower limb, small anteriorly malrotated ears, up-slanting eyes, microophthalmia, a refractive error, a renal anomaly, behavioral problems, and periventricular leukomalacia with mild cerebral atrophy. These features may be a part of various syndromes. CAH was ruled out with laboratory investigations. Table 1 provides a comparison of the features present in the patient with the syndromes associated with each of these features followed by a discussion about each of these syndromes to finally come to the most probable diagnosis.

**Table 1:** Comparison of the features present in the patient. The rows contain possible syndromic differential diagnoses. The first column lists all the features present in the patient. The columns below the name of each syndrome show features present in the patient, which are also found in that particular syndrome.

Features present in the patient ↓	Possible syndromic differential diagnoses and their features →	Congenital adrenal hyperplasia	VATER Syndrome	Townes-Brock syndrome	McKusick-Kaufman syndrome	Bardet-Beidl syndrome
PUGS	-	✔️	✗	✔️	✗	✗
Hydrometrocolpos	-	✗	✗	✗	✔️	✗
Developmental delay/learning difficulties	-	✗	✗	✔️	✗	✔️
Central obesity	-	✗	✗	✗	✗	✔️
Short stature	-	✗	✗	✔️	✗	✗
Post-axial polydactyly (limb abnormality)	-	✗	✔️	✔️	✔️	✔️
Seizures	-	✗	✗	✗	✗	✗
Pendular nystagmus	-	✗	✗	✗	✗	✗
Tapering fingers of the hands (limb abnormality)	-	✗	✔️	✗	✗	✗
Small anteriorly malrotated ears (dysplastic ears)	-	✗	✗	✔️	✗	✗
Upslanting eyes	-	✗	✗	✗	✗	✗
Microphthalmia	-	✗	✗	✗	✗	✗
Refractive errors	-	✗	✗	✗	✗	✔️
Renal anomaly	-	✗	✔️	✔️	✗	✔️
Periventricular leukomalacia with mild cerebral atrophy	-	✗	✗	✗	✗	✗
Behavioral problems	-	✗	✗	✗	✗	✗
Congenital heart disease	-	✗	✔️	✔️	✔️	✗

The patient does not seem to fit in the picture of VATER syndrome (also known as VACTERL association as mentioned before), which is defined as the presence of at least three of the following congenital malformations: vertebral defects, anal atresia, cardiac defects, tracheoesophageal fistula, renal anomalies, and limb abnormalities [11].

Townes-Brock syndrome presents with a classical triad of imperforate anus (84%), dysplastic ears (87%), and thumb malformations (89%). Other features that may be present are renal impairment (42%), including endstage renal disease (ESRD), which may occur with or without structural abnormalities (mild malrotation, ectopia, horseshoe kidney, renal hypoplasia, polycystic kidneys, and vesicoutereral reflux). Congenital heart disease is seen in 25% of those affected. Foot malformations (52%) and genitourinary malformations (36%) are also frequently encountered. Intellectual disability is present in approximately 10% of individuals. Rare features like iris coloboma, Duane anomaly, Arnold-Chiari malformation type 1, and growth retardation may also be present [5,12]. The presence of only one classical feature (dysplastic ears) along with several less common associations makes this an unconvincing diagnosis even though many secondary features are present.

MKS has classical features of postaxial polydactyly (PAP), congenital heart disease (CHD), and HMC in females [6]. Our patient has all three features present. However, there is no proband available within the family. No genetic testing was performed on the patient. The mother has a history of one stillborn child at seven months of gestation before her second pregnancy, but the cause of the stillbirth was not evaluated.

Significant overlap exists between MKS and Bardet-Beidl syndrome [7,9]. Bardet-Beidl syndrome characteristically has rod-cone dystrophy, renal malformations, polydactyly, learning difficulties, central obesity, and hypogonadism [13]. The secondary features are speech disorders or delays, eye abnormalities like strabismus, cataract, and astigmatism, brachydactyly or syndactyly, developmental delay, ataxia, diabetes mellitus, craniofacial dysmorphism, nephrogenic diabetes insipidus, hepatic fibrosis, and left ventricular hypertrophy/congenital heart disease [7,14]. This case could be explained as a presentation of MKS that later developed into Bardet-Beidl syndrome [15]. Significant molecular overlap in addition to phenotypic overlap has been reported to exist among these syndromes, hence making this a plausible possibility [16]. However, the hallmark of Bardet-Beidl syndrome, retinitis pigmentosa, is missing in this case, hence making MKS the most probable diagnosis.

Many of the features that these syndromes encompass need prompt medical care to limit permanent organ damage. For example, PUGS should be corrected as soon as possible to arrest obstructive uropathy and reduce HMC, and sensorineural hearing loss needs to be tended to by the age of six months at the latest to avoid speech delay and hence hinder cognitive development [17]. Endocrinological intervention may help manage short stature. Controlling seizures with anti-seizure medications is essential to prevent further neurological damage. Early stimulation with physiotherapy, enrollment into a special learning school, correction of refractive errors, and diet modifications for obesity are some measures that may help improve the quality of life of such cases.

## Conclusions

PUGS is associated with a limited number of conditions, features of which require prompt diagnosis and corrective intervention. Many children present with phenotypically overlapping syndromic features, hence making a pinpoint diagnosis difficult. This is further complicated in resource-limited settings where physicians may have to rely completely on clinical clues to diagnose complex syndromes. MKS is extremely rare outside the Amish population. Hence, a high index of suspicion for other associated anomalies should be maintained with any presenting congenital anomaly. This may help diagnose syndromes and intervene faster, hence preventing permanent organ damage, limiting disability, and improving the quality of life of affected children.
